# Rituximab with low-dose glucocorticoids for treating refractory membranous nephropathy: a prospective single-center cohort study

**DOI:** 10.3389/fphar.2026.1894444

**Published:** 2026-09-25

**Authors:** Youqi Li, Yu Jiang, Wenwen Jiao, Jianwen Yu, Jianbo Li, Boxun Luo, Junbing He, Wei Chen, Qinghua Liu

**Affiliations:** 1 Department of Nephrology, NHC Key Laboratory of Clinical Nephrology (Sun Yat-Sen University) and Guangdong Provincial Key Laboratory of Nephrology, The First Affiliated Hospital, Sun Yat-sen University, Guangzhou, China; 2 Department of Nephrology, Huizhou Central People’s Hospital, Huizhou, China; 3 Department of Nephrology, The Third Hospital of Changsha, Changsha, China; 4 Department of Nephrology, Jieyang People’s Hospital, Jieyang, China

**Keywords:** clinical remission rate, efficacy, refractory membranous nephropathy, rituximab, safety, steroid

## Abstract

**Background:**

Rituximab (RTX) is the first-line treatment for primary membranous nephropathy (pMN), yet approximately one-third of patients exhibit refractory disease. Evidence supporting RTX combined with low-dose glucocorticoids for refractory pMN remains limited. This study aimed to evaluate the efficacy and safety of this combination regimen in a rituximab-naïve refractory cohort.

**Methods:**

Thirty-six adults with refractory pMN received rituximab plus low-dose glucocorticoids (prednisone equivalent). The primary endpoint was clinical remission at 24 months, analyzed in both the intention-to-treat (ITT, *n* = 36) and per-protocol (PP, *n* = 22) populations. Sensitivity analyses included a worst-case scenario (assuming all dropouts were non-remitters).

**Results:**

Thirty-five patients had evaluable 12-month outcomes, and 26 of them achieved remission (74.3%, with 25.7% achieving complete remission and 48.6% partial remission). By 24 months, the remission rate was 95.5% (21/22) in the per-protocol analysis and 91.67% in the intention-to-treat analysis, compared with 58.3% (21/36) in the worst-case scenario. We found no statistically significant difference in cumulative remission rates between serum PLA2R-positive and PLA2R-negative groups, or between renal tissue PLA2R-positive and PLA2R-negative groups. All 25 patients positive for anti-PLA2R antibodies showed a decreasing antibody trend after treatment, and 80.0% (20/25) achieved complete antibody clearance (antibody titer <20 RU/mL). Adverse events occurred in 37.1% (13/35) of patients, with 11.4% (4/35) of patients developing serious infections. No serious malignant events or fatal events were reported during the study.

**Conclusion:**

In this cohort of patients with refractory pMN, rituximab combined with low-dose glucocorticoids achieved high short-term remission rates, and no safety signals were observed that would preclude further investigation. However, given the uncontrolled study design, repeated RTX dosing, and residual confounding from prior immunosuppression, we cannot draw definitive conclusions on whether adding glucocorticoids offers incremental benefit compared with rituximab monotherapy. The high remission rates observed in this study may also be attributable to repeated rituximab exposure and residual effects of prior immunosuppression. Furthermore, this empirical combination regimen was not designed to test whether glucocorticoids have a direct additive or synergistic effect. Larger randomized controlled trials are therefore warranted to confirm these findings.

## Introduction

Membranous nephropathy (MN) is the leading cause of nephrotic syndrome in adults (Ronco et al., 2021). Over the past decade, the incidence of MN has risen markedly among the Chinese population, and projections indicate that it may soon overtake IgA nephropathy to become the most common glomerular disease (Xu et al., 2016). Approximately one-third of patients with MN may achieve spontaneous remission. However, an estimated 40%–50% of patients with persistent nephrotic syndrome, particularly those with refractory disease, face a risk of progression to end-stage renal disease (ESRD) within 10–15 years (Zhang et al., 2019).

While cyclosporine combined with glucocorticoids can provide short-term relief for patients with IMN, and is considered safe, the relapse rate after discontinuation is as high as 40%–50% (Qiu et al., 2017; Ramachandran et al., 2017; Ferve et al., 2019). Some small observational studies suggest that mycophenolate mofetil (typically combined with prednisone) can effectively reduce proteinuria in patients with IMN (Chan et al., 2007; Choi et al., 2018). However, randomized controlled trials indicate that the 12-month MMF treatment regimen for membranous nephropathy did not reduce the average ratio of proteinuria to creatinine, nor did it increase the rates of partial or complete remission. Furthermore, the incidence of severe adverse reactions remained elevated (Dussol et al., 2008).

Rituximab (RTX), a first-generation anti-CD20 monoclonal antibody, exerts its therapeutic effect by binding to the CD20 antigen on B-cell surfaces, resulting in profound depletion of peripheral blood B lymphocytes and subsequent mitigation of autoimmune responses against self-antigens (Jayne, 2010; Smith et al., 2006; Edwards et al., 2004). The therapeutic potential of RTX in primary membranous nephropathy (pMN) was first reported in 2002, and RTX has demonstrated efficacy in patients unresponsive to angiotensin inhibitors after 6 months of therapy (Remuzzi et al., 2002). Subsequent studies have further validated the efficacy and safety of RTX in managing pMN (Ferve et al., 2019; Fernandez-Juarez et al., 2021; Scolari et al., 2021), leading to its inclusion in the 2021 KDIGO guidelines as a first-line treatment for pMN patients at moderate to high risk of disease progression (KDIGO, 2021). However, 20%–40% of patients still exhibit resistance to rituximab (Ferve et al., 2019; Seitz-Polski et al., 2019). While high-risk patients are typically recommended to receive glucocorticoids combined with cyclophosphamide (CTX) therapy, the significant adverse effects associated with CTX remain a concern (van den Brand et al., 2017).

The optimal management of refractory membranous nephropathy has yet to be determined, as previous studies exploring multitargeted therapies have reported high rates of adverse events (Duan et al., 2023). Recent evidence regarding rituximab–glucocorticoid combinations in primary membranous nephropathy (pMN) remains heterogeneous. A meta-analysis by (Mao et al., 2025) suggested potential efficacy benefits of combination therapy compared with monotherapy, although optimal dosing protocols require further refinement. (Sun et al., 2026) reported that an exposure-optimized rituximab strategy (375 mg/m^2^ on days 1, 15, 30, and 120), when combined with structured prednisone tapering and concentration-guided re-dosing, achieved higher and earlier remission, deeper immunological response, and lower relapse rates than standard rituximab monotherapy.

In contrast, a large retrospective Chinese cohort study (n = 154) by (Qi et al., 2026) found no significant difference in clinical outcomes between rituximab monotherapy and combination regimens, despite the combination group having slightly greater baseline disease severity. These discrepant findings underscore the necessity of prospective data to evaluate rituximab plus low-dose glucocorticoids for refractory pMN, particularly in rituximab-naïve populations. Therefore, this study aimed to evaluate the therapeutic efficacy and safety profile of RTX combined with low-dose glucocorticoids for the treatment of refractory membranous nephropathy.

## Methods

### Study population

This study enrolled patients with biopsy-proven primary membranous nephropathy (PMN) who visited the Nephrology Department of the First Affiliated Hospital of Sun Yat-sen University between August 2019 and August 2023. Patients were included if they met the following criteria: (1) Aged 18–80 years, regardless of gender; (2) Serum albumin ≤30 g/L, 24-h urine protein ≥3.5 g and eGFR (calculated by CKD-EPI formula) ≥ 30 mL/min/1.73 m^2^; (3) Refractory pMN was defined as persistent nephrotic syndrome with unchanged or elevated serum anti-PLA2R antibody levels after more than 6 months of full-dose first-line immunosuppressive therapy, including calcineurin inhibitors, cyclophosphamide, mycophenolate mofetil, tripterygium wilfordii, or leflunomide (Xu et al., 2025). Notably, rituximab-refractory MN was not included in this cohort; (4) Availability of complete clinical data. Patients were excluded if they met any of the following criteria: (1) Secondary membranous nephropathy due to autoimmune diseases, infections, malignancies, or medications; (2) Contraindications to rituximab (e.g., neutrophil count <1.5 × 10^9^/L, platelet count <75 × 10^9^/L, active hepatitis, severe infections, or drug allergies); (3) History of organ transplantation; (4) Concurrent malignancies or rheumatologic autoimmune diseases; (5) Progressive renal failure (creatinine clearance rate <30 mL/min/1.73 m^2^); (6) Pregnancy or lactation; (7) Severe cardiovascular or cerebrovascular diseases, including cerebral infarction, cerebral hemorrhage, myocardial infarction, and heart failure, etc. This prospective, single-center cohort study was initially approved by the Institutional Ethics Committee of the First Affiliated Hospital of Sun Yat-sen University (Approval No. IEC [2019] 482) before patient enrollment commenced. A prespecified study protocol was established before patient enrollment, which defined the treatment regimen (rituximab dosing and initial glucocorticoid therapy), retreatment criteria (based on clinical and serological relapse), core data collection time points, and the initial primary endpoint of 12-month remission rate. The sample size was calculated based on this 12-month endpoint: assuming a 60% historical remission rate with rituximab monotherapy (as reported in the MENTOR trial (Ferve et al., 2019)) and an expected 85% remission rate with combination therapy (derived from preliminary data from our center), 31 evaluable patients were required *a priori* to achieve 80% power at a two-sided significance level of α = 0.05. After accounting for the expected 10% attrition rate, we enrolled a total of 36 patients, all of whom provided signed informed consent. Ultimately, 35 patients had evaluable 12-month outcomes, meeting the original sample-size target. The study was registered with the Chinese Clinical Trial Registry (ChiCTR2000040799) in 2020, after patient enrollment had commenced, and was therefore retrospectively registered. During the registration process, the protocol was revised to extend the primary endpoint to the 24-month remission rate to reflect our intent for long-term follow-up, and a detailed statistical analysis plan (including the use of mixed-effects models) was formally documented in the registry. Under the same assumptions, 31 evaluable patients would also have been required for the 24-month endpoint; however, due to loss to follow-up, only 22 patients had evaluable 24-month outcomes. Therefore, the 24-month analysis was underpowered and should be interpreted with caution.

### Serological assessment of anti-PLA2R antibodies​

Serum anti-phospholipase A2 receptor (PLA2R) IgG antibodies were quantified at baseline (before the first rituximab infusion), month 3, month 6, and every 6 months thereafter, using a commercial ELISA kit (Anti-PLA2R ELISA IgG, EUROIMMUN AG, Lübeck, Germany; catalogue No. EA 1254–9601 G). The assay employs human recombinant M-type PLA2R antigen with horseradish peroxidase-conjugated anti-human IgG, and absorbance is read at 450 nm (reference wavelength 620–650 nm). Samples were diluted 1:101 in sample buffer, and results calculated from a five-point calibration curve were expressed as relative units per milliliter (RU/mL). According to the manufacturer, titres <14 RU/mL indicate negativity, 14–19.9 RU/mL indicate borderline status, and ≥20 RU/mL define seropositivity; the linear range of the assay is 6–1500 RU/mL. For this study, baseline seropositivity was defined as a titre ≥20 RU/mL, antibody clearance was defined as a confirmed decline to below 20 RU/mL, and complete immunological remission was defined as two consecutive measurements <2 RU/mL. Longitudinal changes in titre from baseline through months 3 and 6 were interpreted alongside proteinuria status to guide the decision for a second rituximab course, as detailed in the treatment protocol.

### Treatment protocol

Enrolled patients discontinued their prior immunosuppressive regimens, which included agents such as cyclophosphamide, tripterygium wilfordii, cyclosporine, mycophenolate mofetil (MMF), and tacrolimus. Rituximab (RTX) was administered as two intravenous (IV) infusions of 1,000 mg each, spaced 2 weeks apart. Patients received a second rituximab course at month 6 if they exhibited persistent proteinuria or persistently elevated anti-PLA2R antibodies. Low-dose glucocorticoids were administered at a starting dose of 0.25–0.5 mg/kg/day (prednisone equivalent), adjusted based on the patient’s tolerance and prior medication history. Treatment efficacy was assessed after 1–2 months, and glucocorticoid dosages were tapered according to clinical response. After achieving complete remission, patients underwent a monthly reduction of 5 mg. For those who did not achieve remission within 1–2 months, the initial dose was maintained for a maximum of 3 months before initiating a monthly 5 mg reduction to discontinuation. The total treatment duration did not exceed 1 year (Supplementary Figure 1). Angiotensin-converting enzyme inhibitors (ACEIs) or angiotensin receptor blockers (ARBs) were prescribed to reduce proteinuria and manage blood pressure, tailored to individual patient needs. Statins or ezetimibe were administered to control hyperlipidemia based on baseline lipid profiles. Calcium supplementation and active vitamin D were provided to mitigate the risk of osteoporosis and related complications. Prophylactic antiplatelet or anticoagulant therapy was initiated when indicated to prevent thrombotic events. Additionally, a subset of patients received prophylactic acyclovir or compound sulfamethoxazole based on their clinical risk factors and immune status. Patients positive for hepatitis B core antibodies received entecavir to prevent HBV reactivation. High-risk patients underwent regular monitoring of IgG levels and cytomegalovirus (CMV) DNA via polymerase chain reaction.

Six months after the initial rituximab course, subsequent treatment allocation was determined by a combined assessment of proteinuria and serology. Patients who achieved complete remission (defined as proteinuria ≤0.3 g/24 h) discontinued rituximab, regardless of their anti-PLA2R status. Patients with a partial proteinuria response (urine protein 0.3–3.5 g/24 h, or a reduction of at least 25% from baseline) were eligible for a second two-dose rituximab course. For this group, the final decision also incorporated serial anti-PLA2R kinetics measured between months 3 and 6: patients with an anti-PLA2R titre reduction of less than 50% from baseline or antibody re-elevation received a stronger recommendation for re-dosing, while patients with confirmed antibody clearance (anti-PLA2R < 20 RU/mL) could reasonably defer further treatment. Conversely, patients with an inadequate clinical response (less than 25% reduction in proteinuria) were classified as rituximab-resistant, and rituximab was permanently discontinued regardless of serological trends. Critically, no patient received a second course of treatment based solely on positive anti-PLA2R status without a corresponding proteinuria indication. A small number of patients who were otherwise eligible declined the second course exclusively for financial reasons, and this was prospectively documented.

### Follow-up and monitoring

After the initial administration of rituximab, patients received monthly laboratory assessments, including complete blood count (CBC), serum albumin, serum creatinine, urinalysis, and 24-h urine protein quantification. Comprehensive evaluations were additionally conducted at 3, 6, 9, 12, 18, and 24 months post-treatment. These evaluations covered a wide panel of tests, including CBC, serum albumin, serum creatinine, anti-PLA2R antibody levels, urinalysis, 24-h urine protein quantification, B lymphocyte subset analysis, lipid profile, humoral immunity panel, hepatitis B virus (HBV) serology, HBV DNA quantification, cytomegalovirus (CMV) IgM and IgG antibodies, and CMV DNA quantification.

### Treatment responses and renal outcomes

The primary endpoints were the achievement of partial remission (PR) and complete remission (CR) at 24 months. PR was defined as a urine protein-to-creatinine ratio (UPCR) < 3.0 g/g or a 24-h urine protein excretion <3.0 g/24h, accompanied by a reduction of at least 50% from baseline proteinuria levels. CR was defined as a UPCR <0.3 g/g or 24-h urine protein excretion <0.3 g/24h, paired with a serum albumin level >35 g/L. UPCR measurements were collected from spot urine samples. Patients who did not meet the criteria for either CR or PR were classified as non-responders, while patients who achieved clinical remission were defined as responders. Complete immunological remission was defined as a serum anti-PLA2R level <2.0 RU/mL, and antibody clearance with a level <20 RU/mL was categorized as a partial immunological response.

Secondary endpoints included changes in proteinuria, serum creatinine, serum albumin, total cholesterol, triglycerides, and serum IgG levels from baseline to 1 year after treatment initiation. Additional evaluated outcomes included changes in serum anti-PLA2R antibody titers, relapse rates, and adverse events (AEs). Relapse was defined as a 24-h urine protein excretion that increased to more than 50% above the baseline value, or exceeded 3.5 g/24h in patients who had previously achieved complete remission (CR) or partial remission. Serious adverse events (SAEs) were defined as clinical death or the development of severe complications, including pulmonary infections, pulmonary embolism, cerebral infarction, and myocardial infarction, as well as hospitalization for treatment-related adverse events. While adverse events were collected prospectively, steroid-related toxicities were managed per standard routine clinical practice and were not systematically graded according to CTCAE criteria.

### Statistical analysis

Baseline characteristics are presented as percentages or medians with interquartile ranges (IQRs), and between-group comparisons were performed using the Wilcoxon rank-sum test or Fisher’s exact test when appropriate. We used linear mixed-effects models (LMM) with restricted maximum likelihood (REML) estimation to analyze longitudinal changes in albumin, proteinuria, estimated glomerular filtration rate (eGFR), total cholesterol, triglycerides, serum IgG, CD19^+^ B-cell counts, and anti-PLA2R antibody levels from baseline to the 24-month follow-up. Each model included time (0, 1, 3, 6, 9, 12, 18, and 24 months after treatment initiation) as a categorical fixed effect, and a random intercept for each subject to account for within-subject correlation. Planned contrasts were conducted to compare model-based marginal means between baseline and Month 12, and between baseline and Month 24. Proteinuria, CD19^+^ B-cell counts, and anti-PLA2R antibody levels were log-transformed to satisfy model assumptions; for B-cell counts, the log (x+1) transformation was applied to handle true zero values. Missing data were handled using a REML-based mixed-effects model, which provides valid inference under the assumption that data are Missing At Random (MAR). MAR is an assumption about the data-generating mechanism, not a property intrinsically established by the REML algorithm itself. P-values for all 16 contrasts (8 outcomes × 2 time comparisons) were jointly adjusted using the Benjamini–Hochberg false discovery rate (FDR) procedure. Safety evaluations mainly used descriptive statistics to record the type, frequency, and timing of adverse events. Time-to-event outcomes (remission and relapse) were estimated with the Kaplan-Meier method. We conducted sensitivity analyses including intention-to-treat (ITT), per-protocol, and worst-case analyses (where all dropouts were defined as non-remitters). Subgroup analyses stratified by the number of rituximab treatment courses were also performed, with time intervals calculated from the date of the first rituximab infusion. Statistical significance was defined as a two-sided P-value <0.05. All statistical analyses were strictly performed according to this pre-specified SAP, with no additional *post hoc* modifications. All statistical analyses were performed using R version 4.6.1 with the lme4 and emmeans packages, alongside Free Statistics software version 1.8.

## Results

### Baseline characteristics

This study enrolled 36 patients who provided signed informed consent. One patient was lost to follow-up at month 7 and was excluded from all loss-to-follow-up analyses. The remaining 35 patients were included in the analysis. Among these 35 patients, 13 were lost to follow-up at different time points during the study. The main reasons for loss to follow-up were disruptions related to the COVID-19 pandemic (n = 9; patients were unable to travel or access medical care) and transfer to other medical institutions (n = 4), with a median time to loss to follow-up of 15 (IQR: 12–18) months. A flow chart of the study is provided in Figure 1. The median age of participants was 52 years, with a male predominance (25 males, 71.4%; 10 females, 28.6%). The median disease duration was 24 months. Of the 35 patients, 34 (97.1%) reported a history of glucocorticoid use, and 24 (68.6%) had received immunosuppressive therapy within 3 months before enrollment. Previous therapeutic regimens included tacrolimus (22 patients), cyclosporine (18 patients), cyclophosphamide (9 patients), Tripterygium wilfordii (5 patients), mycophenolate mofetil (MMF, 4 patients), and leflunomide (2 patients) (Table 1). Calcineurin inhibitors (CNIs) were the most commonly used prior agents, administered to 28 patients (80.0%) with a median treatment duration of 9 months. Specifically, 22 patients received tacrolimus (median dose 2.0 mg/d; median washout period 4.0 weeks) and 18 received cyclosporine (median dose 175 mg/d; median washout period 11.0 weeks). Notably, among patients treated with CNIs, the best response was partial remission (PR) in 42.9% (12/28) of cases and no response (NR) in 50.0% (14/28), which highlights the refractory nature of this patient cohort. Nine patients (25.7%) received cyclophosphamide treatment with a median duration of 6.0 months (total dose 0.8 g per month). Among these patients, 44.4% achieved PR and 50% had NR. Four patients (11.4%) received mycophenolate mofetil (MMF) at a median dose of 2.0 g/d for a median treatment duration of 8.0 months, and all four showed no response. Other prior treatments included Tripterygium wilfordii (n = 5) and leflunomide (n = 2). Washout periods varied considerably across different treatment regimens. While the median interval between CNI discontinuation and rituximab initiation was 4–11 weeks, cyclophosphamide and Tripterygium wilfordii had significantly longer washout intervals (median 48.0 weeks and 56.0 weeks, respectively). This pattern likely reflects both the cumulative toxicity profiles of these agents and the timing of disease relapse relative to drug cessation (Supplementary Table 1).

**FIGURE 1:**
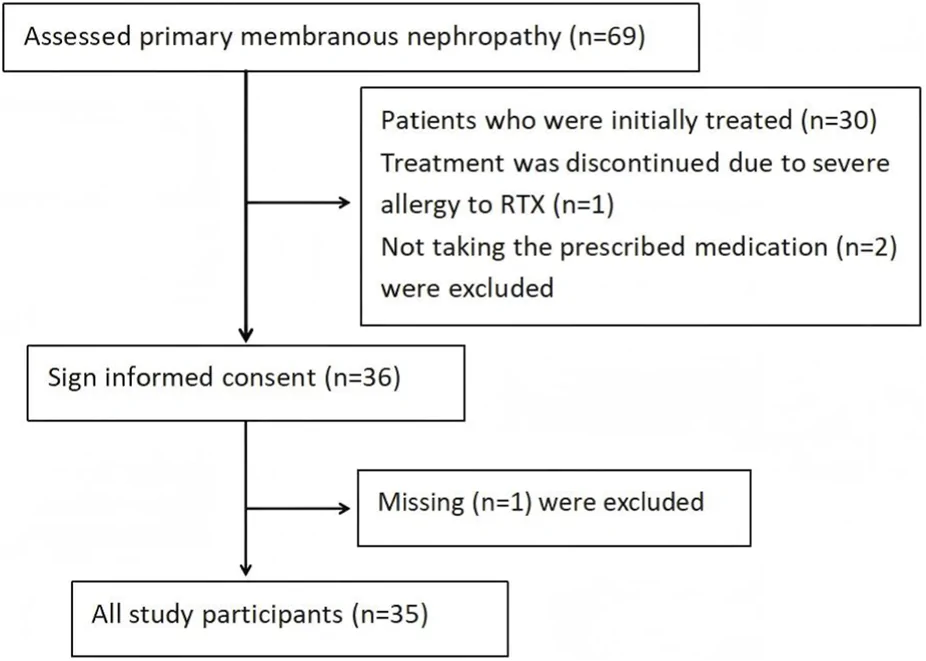
Flowchart of the study.

**TABLE 1:** Baseline characteristics of patients with PMN included in this study.

Index	Total (n = 35)
Age (years)	52.0 (39.5, 57.0)
Gender [n (%)]	
Male	25 (71.4%)
Female	10 (28.6%)
BMI (kg/m^2^)	22.4 (20.6, 24.0)
Disease duration (months)	24.0 (12.0, 38.5)
MAP (mmHg)	96 (85.8, 102)
History of glucocorticoid use	34 (97.1%)
Immunosuppressive treatment within 3 months	24 (68.6%)
Previous use of immunosuppressants	
Tacrolimus	22 (62.9%)
Cyclosporine	18 (51.4%)
Cyclophosphamide	9 (25.7%)
Tripterygium wilfordii	5 (14.3%)
Mycophenolate mofetil	4 (11.4%)
Leflunomide	2 (5.7%)
Haemoglobin (g/L)	120.0 (110.5, 132.5)
White blood cell count (×10^9^/L)	8.9 (6.9, 10.2)
Lymphocyte (×10^9^/L)	2.2 (1.9, 2.9)
Albumin (g/L)	20.6 (18.0, 24.2)
Cholesterol (mmol/L)	6.8 (5.2, 8.2)
LDL-c (mmol/L)	4.1 (3.3, 4.9)
HDL-c (mmol/L)	1.7 (1.2, 2.0)
Triglycerides (mmol/L)	2.0 (1.6, 2.5)
Serum creatinine (μmol/L)	91.0 (70.0, 113.5)
eGFR (mL/min/1.73 m^2^)	77.2 (59.6, 105.7)
eGFR< 60 mL/min/1.73 m^2^ [n (%)]	9 (25.7%)
Proteinuria (g/24h)	6.7 (5.1, 9.7)
IgA (g/L)	1.4 (1.1, 2.0)
IgM (g/L)	0.9 (0.6, 1.2)
IgG (g/L)	4.0 (2.7, 4.7)
C3 (g/L)	0.9 (0.8, 1.0)
C4 (g/L)	0.2 (0.2, 0.2)
Glomerular PLA2R positivity [n (%)]	30 (85.7)
Serum anti-PLA2R titer (RU/mL)	60.0 (20.0, 100.0)
Anti-PLA2R antibodies (>150 RU/mL) [n (%)]	2 (5.7%)
Anti-PLA2R antibody positivity [n (%)]^a^	25 (71.4%)
Absolute values of CD19 (μL)	240.6 (143.0, 414.4)
Baseline glucocorticoid dose (mg/d)	30.0 (25.0, 30.0)
Cumulative steroid exposure (g)	9.5 (8.6–10.6)

Data are median (25th, 75th percentiles), or n (%). Where not specified, the available n is the same as the total number of cases in the top row.

Abbreviations: BMI, body mass index; MAP, mean arterial pressure; LDL-c, low density lipoprotein cholesterol; HDL-c, high density lipoprotein cholesterol; eGFR, estimated glomerular filtration rate; C3, complement 3; C4, complement 4; PLA2R, phospholipase A2 receptor; SAb, Serum anti-PLA2R antibody; a, Anti-PLA2R, positivity was defined by a value > 20 RU/mL.

At baseline, the median proteinuria level was 6.7 (IQR: 5.1, 9.7) g/24 h, serum albumin was 20.6 (IQR: 18.0, 24.2) g/L, serum creatinine was 91.0 (IQR: 70.0, 113.5) μmol/L, total cholesterol was 6.8 (IQR: 5.2, 8.2) mmol/L, and eGFR was 77.2 (IQR: 59.6, 105.7) mL/min/1.73 m^2^. All 35 patients underwent anti-PLA2R antibody testing, which yielded a median antibody level of 60.0 (IQR: 20.0, 100.0) RU/mL, with 25 patients (71.4%) testing positive (>20 RU/mL). Among 30 patients tested for PLA2R in renal biopsy tissue, the positivity rate was 85.7%. Five patients (14.3%) tested negative for both serum anti-PLA2R antibodies and renal tissue PLA2R. The median baseline glucocorticoid dosage was 30.0 (IQR: 25.0, 30.0) mg/d (Table 1). Compared to patients achieving clinical remission, nonresponders exhibited significantly elevated levels of total cholesterol, LDL-c, and HDL-c, but demonstrated markedly lower serum IgG levels (Table 2).

**TABLE 2:** Composite comparisons of clinical features of patients with PMN between responders and nonresponders at baseline.

Index	Responders (n = 26)	Nonresponders (n = 9)	*P* value
Age (years)	54.0 (44.8, 57.0)	40.0 (39.0, 52.0)	0.085
Gender [n (%)]	​	​	0.694
Male	19 (73.1%)	6 (66.7%)	​
Female	7 (26.9%)	3 (33.3%)	​
BMI (kg/m^2^)	22.1 (20.5, 24.1)	22.8 (22.0, 23.6)	0.792
MAP (mmHg)	96.1 (86.5, 101.7)	93.5 (91.2, 100.7)	0.91
Albumin (g/L)	21.2 (18.9, 26.8)	18.1 (17.2, 23.1)	0.199
Cholesterol (mmol/L)	6.5 (5.0, 7.2)	8.3 (7.3, 8.8)	0.009
LDL-c (mmol/L)	3.9 (3.2, 4.5)	5.4 (5.0, 7.7)	0.003
HDL-c (mmol/L)	1.6 (1.2, 1.9)	2.0 (1.7, 4.3)	0.038
Triglycerides (mmol/L)	1.9 (1.6, 2.3)	2.4 (2.0, 3.0)	0.096
Serum creatinine (μmol/L)	92.5 (79.0, 112.8)	84.0 (58.0, 114.0)	0.485
eGFR (ml/min/1.73 m^2^)	76.5 (56.7, 99.2)	107.1 (62.1, 107.7)	0.242
Proteinuria (g/24h)	6.4 (5.1, 8.6)	11.3 (5.6, 11.8)	0.131
Ig A (g/L)	1.3 (1.0, 1.9)	1.6 (1.4, 2.3)	0.151
Ig M (g/L)	0.9 (0.6, 1.3)	0.9 (0.8, 1.0)	0.763
Ig G (g/L)	4.3 (3.4, 5.0)	2.6 (2.2, 3.4)	0.003
C3 (g/L)	0.8 (0.7, 1.0)	1.0 (0.9, 1.1)	0.049
C4 (g/L)	0.2 (0.2, 0.2)	0.2 (0.2, 0.3)	0.468
Absolute values of CD19 (uL)	231.3 (188.3, 393.6)	244.7 (138.9, 489.8)	0.776
Serum anti-PLA2R titer (RU/mL)	60.0 (20.0, 93.8)	30.0 (25.4, 112.5)	0.732
Glomerular PLA2R positivity [n (%)]	23 (88.5%)	7 (77.8%)	0.586
Cumulative steroid exposure (g)	5.1 (4.5, 5.4)	4.3 (3.0,4.5)	0.016

Data are median (25th, 75th percentiles), or n (%). Where not specified, the available n is the same as the total number of cases in the top row.

Abbreviations: BMI, body mass index; MAP, mean arterial pressure; LDL-c, low density lipoprotein cholesterol; HDL-c, high density lipoprotein cholesterol; eGFR, estimated glomerular filtration rate; C3, complement 3; C4, complement 4; SAb, Serum anti-PLA2R antibody; Values in bold represent P < 0.05.

### Longitudinal changes in clinical parameters over 24 months

Significant changes in laboratory parameters were observed after treatment. At the 12-month follow-up, we detected marked elevations in serum albumin [20.6 (IQR: 17.9, 24.5) vs. 37.9 (IQR: 31.8, 40.3) g/L, *P* < 0.0001] and immunoglobulin G (IgG) levels [3.99 (IQR: 2.65, 4.69) vs. 7.52 (IQR: 4.68, 9.38) g/L, *P* < 0.0001], alongside substantial reductions in serum total cholesterol [6.80 (IQR: 5.20, 8.30) vs. 5.05 (IQR: 4.18, 5.95) mmol/L, *P* < 0.0001], triglycerides [2.05 (IQR: 1.62, 2.50) vs. 1.31 (IQR: 1.45, 1.91) mmol/L, *P* = 0.001], and 24-h urinary protein excretion [6.68 (IQR: 4.97, 9.84) vs. 1.34 (IQR: 0.32, 3.79) g/24h, *P* < 0.0001]. Notably, estimated glomerular filtration rate (eGFR) remained stable with no significant variation [77.20 (IQR: 58.13, 106.30) vs. 81.78 (IQR: 68.94, 99.52) mL/min/1.73 m^2^, *P* = 0.4167] (Figure 2). Linear mixed-effects model analyses with FDR adjustment further supported these observations, confirming that all improvements except triglycerides remained statistically significant at the 24-month follow-up (pFDR <0.02 for all other comparisons). Consistent with the 12-month findings, eGFR showed no significant change at either 12 months (pFDR = 0.47) or 24 months (pFDR = 0.08).

**FIGURE 2:**
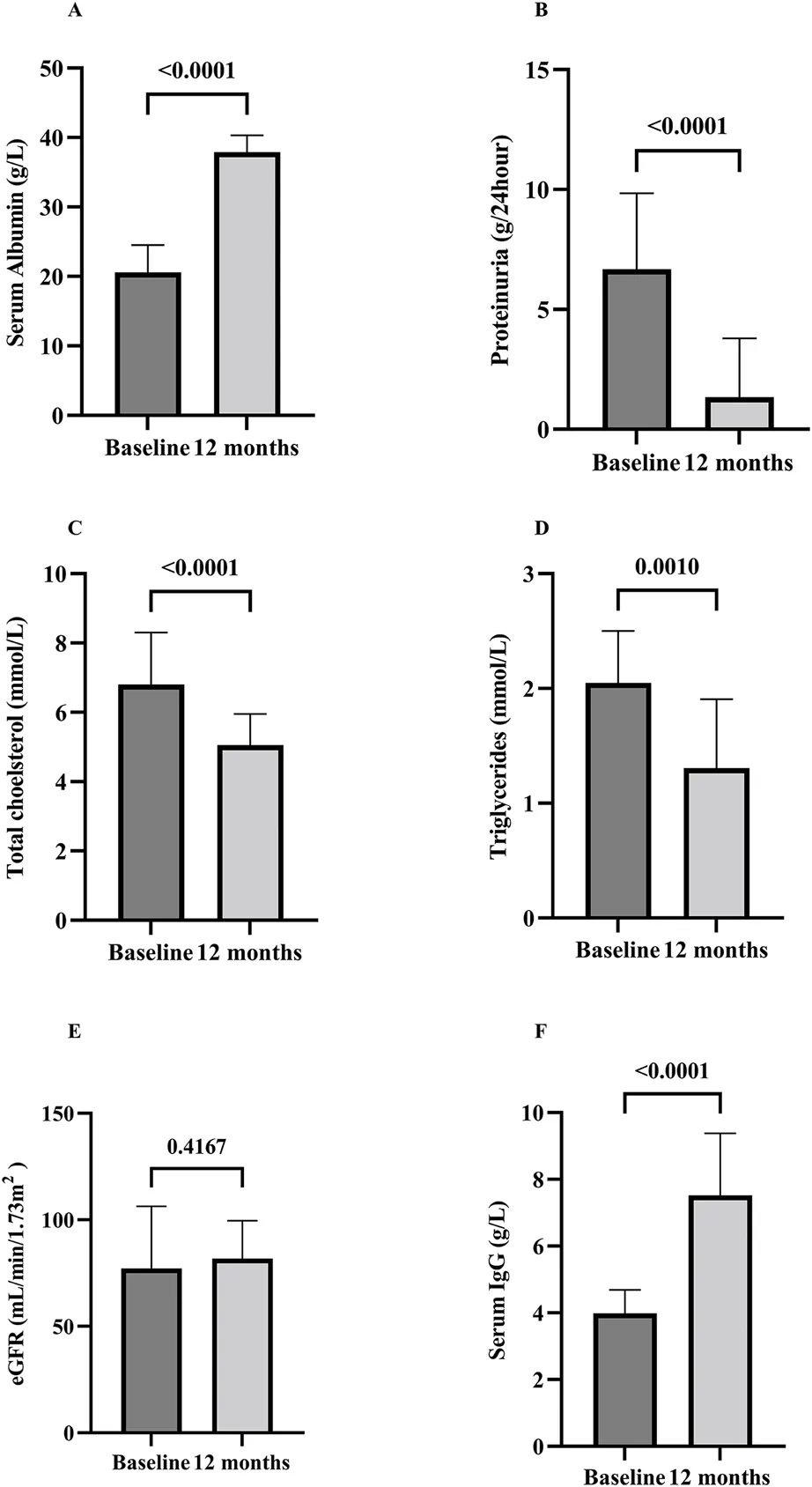
Comparison of clinical parameters after 12 months of treatment. **(A)** Albumin; **(B)** 24-h proteinuria; **(C)** total cholesterol; **(D)** triglycerides; **(E)** eGFR; **(F)** serum IgG level. Paired data at baseline and during follow-up were compared using the t-test for normally distributed data or Mann-Whitney U test for nonparametric distribution. Gray bars represent data before treatment, while the white bars represent data 12 months after treatment. The bars show the medians and interquartile ranges (IQRs) for each variable.

These significant improvements in laboratory parameters were sustained throughout the 24-month follow-up, with continued favorable changes seen in key clinical outcomes. Over the full 24-month observation period, patients with primary membranous nephropathy (pMN) maintained consistent therapeutic responses. We observed progressive reductions in anti-PLA2R antibody titers (from a median of 60.0 [IQR: 20.0–100.0] RU/mL to 5.0 [IQR: 5.0–6.29] RU/mL), 24-h urinary protein excretion (from 6.7 [IQR: 5.1–9.7] g/24h to 1.3 [IQR: 0.3–3.8] g/24h), and absolute CD19^+^ B-cell counts, which were paralleled by steady increases in serum albumin concentrations (from 20.6 [IQR: 18.0–24.2] g/L to 37.9 [IQR: 31.8–40.3] g/L) (Figure 3). Mixed-model estimates further indicated that at 24 months, anti-PLA2R antibodies and CD19^+^B-cell counts remained persistently reduced (anti-PLA2R: estimate = −1.81 log-units, 95% CI: 2.24 to −1.39, pFDR <0.001; CD19^+^ B-cell: 2.25 log-units, 95% CI: 2.86 to −1.64, pFDR <0.001), alongside sustained elevation of albumin (+15.14 g/L, 95% CI: 13.19 to 17.10, pFDR <0.001) and sustained reduction of proteinuria (−2.46 log-units, 95% CI: 2.82 to −2.09, pFDR <0.001) (Supplementary Figure 1).

**FIGURE 3:**
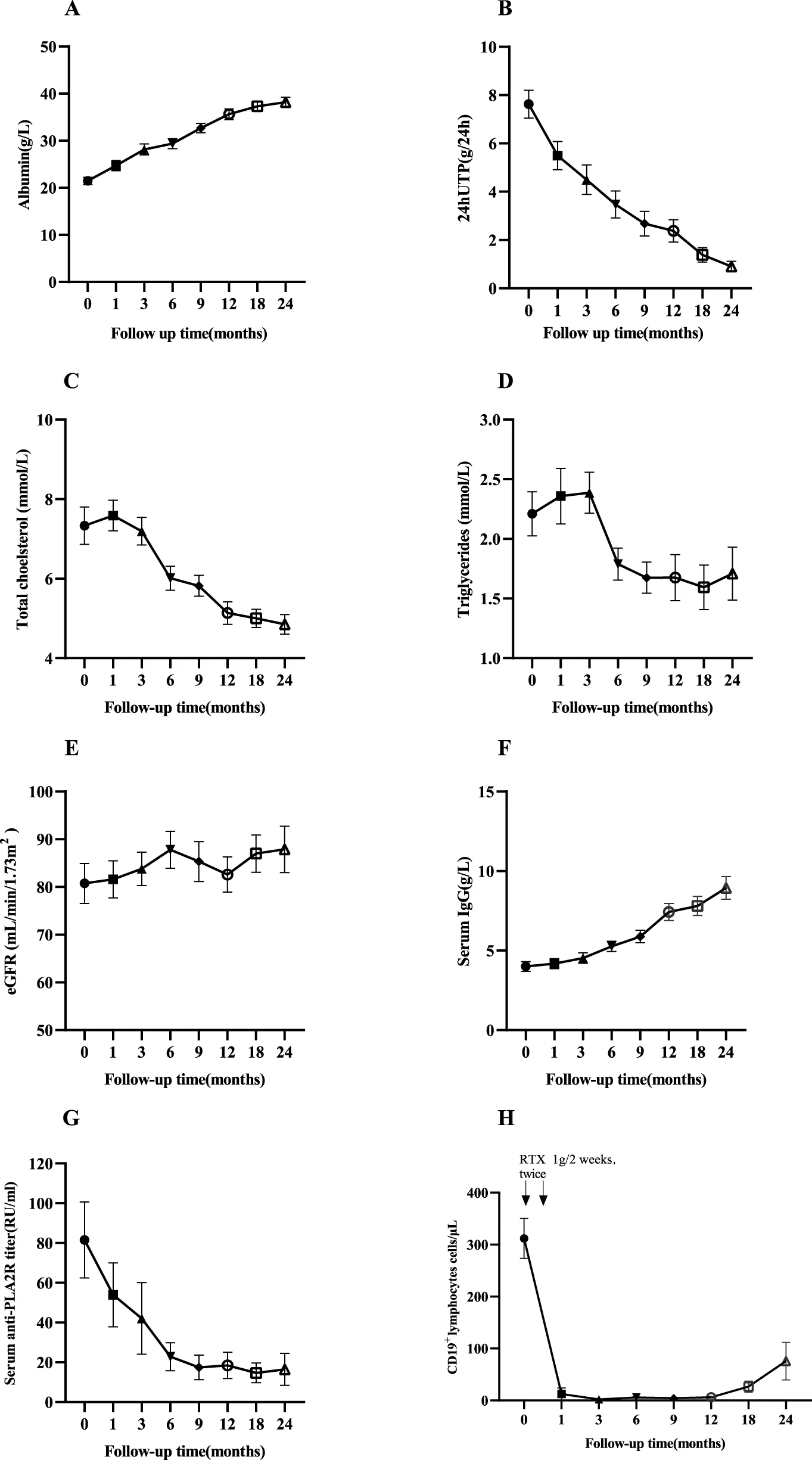
Changes in laboratory parameters after rituximab treatment in patients who had been followed up for 24 months. **(A)** Albumin; **(B)** 24-hour proteinuria; **(C)** total cholesterol; **(D)** triglycerides; **(E)** eGFR; **(F)** serum IgG level; **(G)** Anti-PLA2R antibody titer; **(H)** The absolute values of CD19. The bars show the SEM value of the mean for each variable.

### Efficacy assessment

We enrolled 36 patients in this study, all of whom provided signed informed consent. Thirty-five of these participants completed at least 12 months of follow-up and constituted the 12-month evaluable population, while all 36 patients were included in the intention-to-treat (ITT) analysis. Of these, 22 patients reached 24 months and formed the per-protocol (PP) cohort. At the 12-month time point, 26 of the 35 evaluable patients (74.3%) achieved a clinical response, including complete remission (CR, n = 9, 25.7%) and partial remission (PR, n = 17, 48.6%) (Figures 4A,C). Applying an intention-to-treat (ITT) approach and conservatively assigning patients lost to follow-up as non-responders (n = 36), the overall response rate was 72.2% (95% CI: 54.8%–86.0%), consistent with the prespecified primary analysis (Figure 4B). Among the 22 patients with available 24-month data, 21 (95.5%) had entered remission (11 CR, 50.0%; 10 PR, 45.5%), representing a complete-case analysis restricted to participants who completed the entire study protocol. In sensitivity analyses based on the full ITT cohort (n = 36), the 24-month remission probability estimated by the Kaplan-Meier method was 91.67% (95% CI:72.3%–99.2%), while a worst-case scenario assuming all dropouts as non-remitters yielded a remission rate of 58.3% (21/36). The marked difference among these three estimates (95.5%, 91.67%, and 58.3%) clearly illustrates the inherent uncertainty due to attrition bias.

**FIGURE 4:**
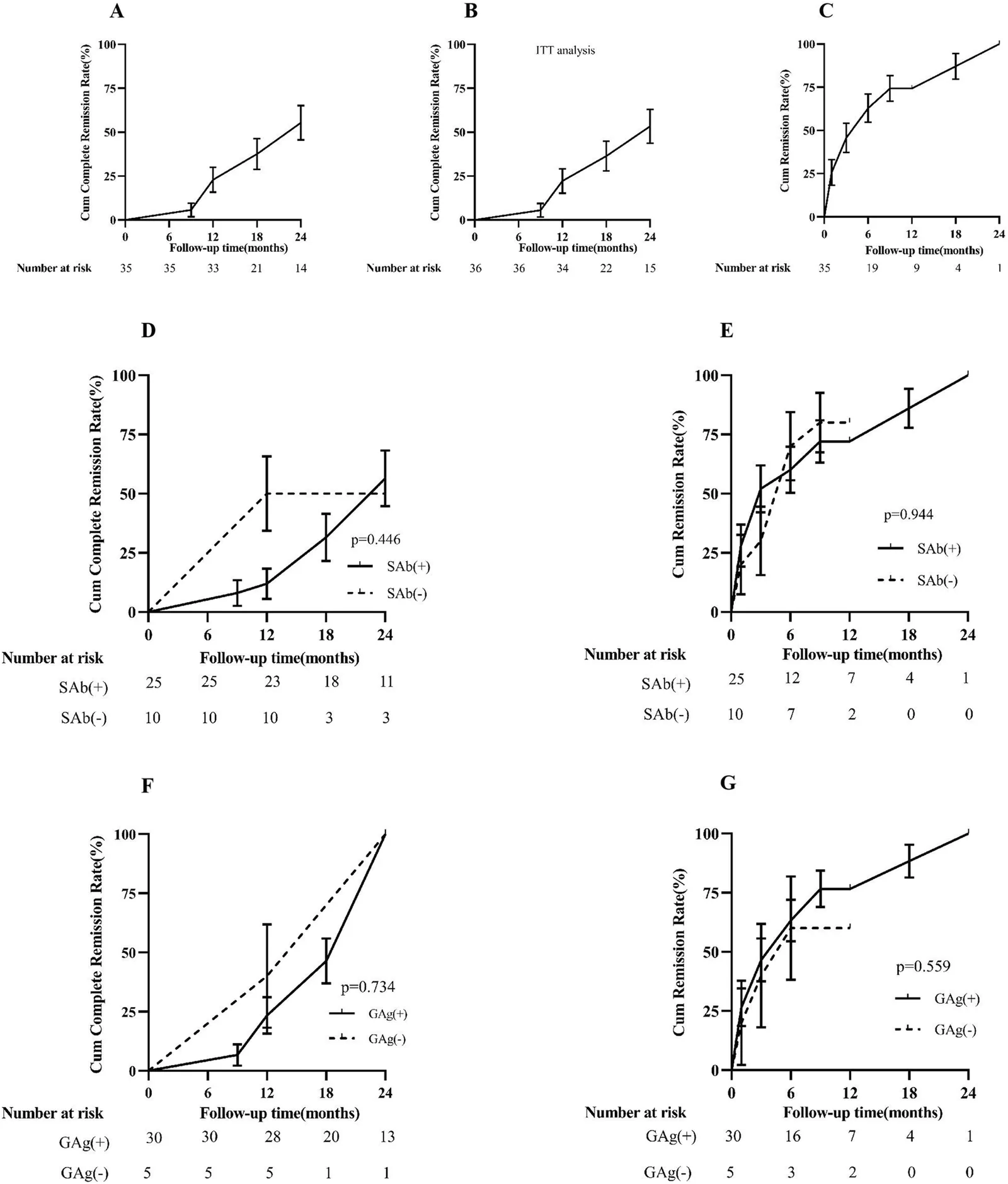
Kaplan ‐Meier estimates of the composite remission (complete or partial) after rituximab treatment in patients who had been followed up for 24 months. **(A)** Accumulative complete remission (CR) rate after RTX treatment; **(B)** ITT analysis of CR; **(C)** Accumulative remission rate after RTX treatment; **(D)** Comparison of accumulative CR rate in the SAb+ group and SAb− group, P = 0.446; **(E)** Comparison of the accumulative remission rate in the SAb+ group and SAb− group, P = 0.944; **(F)** Comparison of accumulative CR rate in the Glomerular PLA2R+ group and Glomerular PLA2R− group, P =0.734; **(G)** Comparison of accumulative remission rate in the Glomerular PLA2R+ group and Glomerular PLA2R− group, P = 0.559.

Twenty-three patients received a second course of rituximab at month 6. Compared with baseline, this cohort showed a marked reduction in anti-PLA2R antibody levels (median 60 [IQR: 20, 100] vs. 7.22 [IQR: 5.17, 11.45] RU/mL, *P* = 0.002, Supplementary Figure 2), and had a significantly higher 12-month response rate than patients who received only a single course (87.0% [20/23] vs. 50.0% [6/12], P = 0.037, Supplementary Table 2). Of the 12 patients who remained on a single course, half (n = 6) presented with both clinical and immunological quiescence at month 6 and required no further treatment; the remaining 6 met retreatment criteria for persistent proteinuria with non-declining PLA2R antibody titers, but declined a second course solely for financial reasons. No patient received a second course based only on persistent anti-PLA2R antibodies, which is consistent with our proteinuria-first retreatment algorithm. Only one patient (2.9%) experienced relapse 18 months after achieving complete remission; re-treatment with RTX (1,000 mg × 2 doses, administered 2 weeks apart) induced remission again.

Among the 25 baseline patients with positive anti-PLA2R antibodies, antibody titers significantly decreased after RTX treatment, with 20 cases (80.0%) achieving antibody clearance (defined as <20 RU/mL) and 4 cases (16.0%) achieving complete immunological remission (defined as two consecutive <2 RU/mL per KDIGO-aligned threshold). The clinical remission rate in this seropositive cohort was 84% (21 of 25), compared to 80% (8 of 10) in the seronegative group. Complete remission rates were 44% (11 of 25) and 50% (5 of 10) in the seropositive and seronegative groups, respectively. No statistically significant differences were observed in clinical or complete remission rates between groups (P = 0.944 and P = 0.446, respectively) (Figures 4D,E). Reflecting the refractory nature of the cohort, baseline anti-PLA2R titres were heterogeneous (median 60 [IQR 20–100] RU/mL), with many patients showing plateau rather than sharply rising trajectories under prior non-B-cell therapy. Among seropositive responders, median time to antibody clearance was 3.0 (IQR 1.0–6.5) months; in this subgroup, immunological remission preceded clinical remission by a median of 3.5 (IQR 2.8–5.3) months. On histopathology, 30 patients were tissue PLA2R-positive, with a remission rate of 83.3% (25/30), versus 60.0% (3/5) in tissue-negative cases (P = 0.559). Given the limited number of PLA2R-negative participants, efficacy in this subset remains exploratory (Figures 4F,G).

### Subgroup analyses

Exploratory subgroup analyses were conducted to investigate whether prior non-B-cell-targeted immunosuppression affected response to rituximab-steroid therapy. Remission rates were broadly similar between patients who had previously received individual immunosuppressive agents and those who had not: cyclosporine (66.7% [12/18] vs. 70.6% [12/17]), tacrolimus (72.7% [16/22] vs. 61.5% [8/13]), cyclophosphamide (77.8% [7/9] vs. 69.2% [18/26]), and mycophenolate mofetil (50.0% [2/4] vs. 64.5% [20/31]). Similarly, remission rates did not differ significantly between patients who received immunosuppression within 3 months before enrollment and those who underwent a longer washout period (66.7% [16/24] vs. 72.7% [8/11]). None of these between-group differences reached statistical significance (all P > 0.05).

Beyond prior baseline exposure, we further investigated whether the protocol-specified second course of rituximab influenced long-term outcomes. We conducted a post-hoc subgroup analysis comparing patients who declined a second course due to financial constraints (n = 6) with those who received the pre-specified second course (n = 23). Detailed comparative results are presented in Supplementary Table 3. In short, the 12-month overall remission rates were comparable between the two groups (83.3% vs. 78.3%). However, among patients who completed 24-month follow-up, only 50.0% (2/4) of those who declined retreatment maintained remission, compared with 94.4% (17/18) in the retreated cohort.

### Adverse events

Adverse events (AEs) were reported in 13 patients (37.1%), and 4 patients (11.4%) experienced serious adverse events (SAEs). All percentages are calculated on a per-patient basis. All SAEs were infection-related, comprising one case of cytomegalovirus (CMV) infection and three cases of pulmonary infection. These events occurred despite the implementation of standardized prophylactic measures, including entecavir prophylaxis for patients positive for hepatitis B core antibody (HBcAb), trimethoprim-sulfamethoxazole prophylaxis against Pneumocystis jirovecii pneumonia, and regular serial monitoring of IgG and CMV-DNA via PCR for high-risk individuals. Hospitalization durations for the four patients with SAEs were 8, 8, 9, and 10 days, respectively; all patients achieved full recovery and were discharged after targeted intravenous anti-infective and antiviral therapy (Table 3). Infusion-related reactions (e.g., rash, pruritus, gastrointestinal symptoms, tachycardia, and transient blood pressure fluctuations) were effectively managed by reducing the infusion rate or temporarily pausing infusion, and did not recur with subsequent administrations. Notably, chart reviews revealed no instances of glucocorticoid-induced psychosis, peptic ulcer disease, or avascular necrosis. Furthermore, no obesity or osteoporosis-related events were observed, likely attributable to the relatively low cumulative corticosteroid dose and the 24-month follow-up duration.

**TABLE 3:** Adverse events in all patients with PMN receiving rituximab.

Adverse events	Our study (n = 35)		Mentor study (n = 65)	
	No. of events	Patients [n(%)]	No. of events	Patients [n(%)]
Adverse events	18	13 (37.1%)	179	46 (70.8%)
Infusion reactions^*^	7	7 (20.0%)	22	16 (24.6%)
**Infection**	11	9 (25.7%)	22	16 (24.6%)
Upper respiratory infection	3	2 (5.7%)	12	9 (13.8%)
Skin, subcutaneous bacterial infections	3	2 (5.7%)	5	4 (6.2%)
Urinary tract infection	1	1 (2.9%)	—	—
Pneumonia	3	3 (8.6%)	1	1 (1.5%)
Cytomegalovirus infection	1	1 (2.9%)	—	—

*Infusion reactions include rash, erythema, pruritus, runny nose, and irritability.

## Discussion

In this prospective, single-center study that enrolled 35 consecutive patients with primary refractory membranous nephropathy, we evaluated 2-year outcomes of a treatment regimen combining rituximab and low-dose glucocorticoids (combination therapy). Our key findings are as follows: First, the overall response rate for primary refractory membranous nephropathy treated with this regimen reached 74.3% at 12 months, and increased to 95.5% by 24 months. Renal function remained relatively stable throughout the study, and no patients progressed to end-stage renal disease (ESRD). Second, 80% of serum anti-PLA2R antibody-positive patients achieved immunological remission. Subgroup analyses showed no significant differences in complete or overall response rates between the serum antibody-negative and antibody-positive groups. Similarly, no statistically significant differences in overall efficacy or complete response rates were detected between the tissue PLA2R-positive and negative groups. Third, during the follow-up period, four patients developed severe adverse events, and all made a full recovery after hospitalization. Nevertheless, given that all these events were infection-related, the overall safety profile of this regimen should be interpreted with caution.

This study demonstrated the efficacy of rituximab (RTX) combined with low-dose glucocorticoids for the treatment of primary refractory membranous nephropathy. Our results showed an overall remission rate of 74.3% at 12 months, which rose to 95.5% by 24 months. We cannot definitively attribute these observed remission rates to the addition of low-dose glucocorticoids, as the absence of a contemporaneous RTX monotherapy control group prevents us from drawing causal conclusions about the incremental benefit of combining glucocorticoids. The remission rates reported in our study are higher than those previously documented in earlier studies investigating the efficacy of first-line RTX monotherapy for membranous nephropathy (Ferve et al., 2019; Scolari et al., 2021; Ruggenenti et al., 2003; Ruggenenti et al., 2012; Dahan et al., 2017; Yan et al., 2024; Zhang et al., 2023). Notably, research on the use of RTX specifically for refractory membranous nephropathy remains limited. In a retrospective study by (Wang et al., 2018), the 12-month remission rate for patients with refractory membranous nephropathy treated with RTX was only 41.7%. The discrepancy in remission rates between our study and previous work may be explained by several factors. First, our treatment protocol combined RTX with low-dose glucocorticoids. As a cornerstone of immunotherapy for idiopathic membranous nephropathy, glucocorticoids exert broad anti-inflammatory effects. This potential mechanism may partly explain the observed remission in our cohort, although the absence of a rituximab-monotherapy comparator prevents us from determining whether glucocorticoids provided an incremental benefit. Second, this study adopted a standardized two-dose RTX regimen. In contrast, only 41.6% of patients in Wang et al.‘s study received the standard two-dose regimen, with the remaining patients treated under a B cell-guided protocol that used lower RTX doses (Wang et al., 2018). Existing evidence confirms that higher RTX doses are associated with improved remission rates (Seitz-Polski et al., 2019; Moroni et al., 2017). Third, two-thirds of our patients received immunosuppressive therapy within 3 months prior to enrollment, and their median anti-PLA2R antibody levels were lower than those reported in some randomized controlled trials investigating RTX treatment for MN (e.g., the MENTOR trial: 409 RU/mL). Therefore, the residual effect of prior immunosuppressive therapy may have contributed to the improved therapeutic efficacy observed here. Fourth, the baseline estimated glomerular filtration rate (eGFR) of participants in this study was above 30 mL/min, indicating relatively preserved renal function. Existing studies have shown that patients with severe tubulointerstitial injury and impaired renal function are less likely to respond favorably to immunotherapy, and also tend to have a diminished response to RTX (KDIGO, 2021). This association may be mechanistically linked to urinary rituximab loss: recent evidence directly links heavy proteinuria and substantial urinary IgG excretion to increased RTX clearance via the urine, which may blunt systemic drug exposure and raise the risk of treatment failure in membranous nephropathy (Allinov et al., 2025). In our cohort, baseline proteinuria was accompanied by preserved renal function and a relatively favourable selectivity pattern, and both urinary IgG loss and proteinuria declined rapidly after treatment—factors likely preserving effective RTX exposure and contributing to the observed efficacy (Allinovi et al., 2022).

RTX therapy has been proven to effectively eliminate anti-PLA2R antibodies, which are key biomarkers for membranous nephropathy (Ruggenenti et al., 2017; Hoxha et al., 2014; Ruggenenti et al., 2015). Previous studies have consistently found that anti-PLA2R antibody levels decrease significantly in seropositive patients 1 year after RTX treatment. Our findings are consistent with these observations: we observed a marked reduction in PLA2R antibody levels in seropositive patients after RTX administration, with complete antibody clearance achieved in 80.0% of cases. Notably, Remuzzi (Remuzzi et al., 2002) and other researchers have pointed out that patients with high antibody titers have considerably lower clinical remission rates than those with low antibody titers. (Ruggenenti et al., 2012) further clarified that baseline anti-PLA2R antibody levels can predict the clinical outcomes of patients receiving RTX therapy: antibody titers increase during periods of disease activity and decrease before clinical remission is achieved. More importantly, persistent positivity for PLA2R antibodies has been identified as an independent risk factor for non-remission after RTX treatment. As a result, anti-PLA2R antibody levels are generally regarded as a reliable biomarker for evaluating the therapeutic efficacy of RTX (Ferve et al., 2019). Nevertheless, our analysis showed no significant difference in cumulative clinical remission rates between patients who were PLA2R antibody positive at baseline and those who were negative. This inconsistency may be explained by two reasons. First, all participants in our study had received prior immunosuppressive therapy, which may have changed serum PLA2R levels, making them different from the baseline levels measured at disease onset. Second, the limited sample size of our study reduced the statistical power of our analysis.

Our findings underscore that the therapeutic efficacy of RTX for primary membranous nephropathy is contingent upon treatment duration. Patients who had not achieved clinical remission by the sixth month after treatment showed gradual improvement over time, with some attaining clinical remission later. In this study, seven patients classified as nonresponders at the six-month mark achieved partial remission by the 18th month after additional RTX treatments were discontinued, while they continued taking oral corticosteroids. Notably, one patient achieved complete remission by the 24th month. However, due to limited follow-up duration, only 22 cases reached the 24-month endpoint, so extending the observation period could potentially increase the remission rate. Additionally, we observed that serum antibodies became undetectable and B-cell regeneration started after 18 months. Adjusting subsequent RTX treatments based on B-cell counts may further improve clinical remission rates.

In our refractory cohort, baseline anti-PLA2R antibody status alone could not predict treatment response; instead, the kinetic trend of antibody levels was far more predictive than baseline titer. Many patients receiving salvage therapy already had partially suppressed titers from prior immunosuppression, which explains why static baseline measurements failed to stratify clinical outcomes. A clear kinetic pattern emerged: patients whose antibody titers did not decrease by month 3, or remained elevated at month 6, typically required a second course of rituximab. This clinical observation was supported by our comparative outcome data. Although initial remission rates were comparable between groups, the durability of response differed markedly: at 24 months, only 50% of patients who declined a second course maintained remission, compared with 94.4% of patients who received retreatment. This notable discrepancy suggests that repeated B-cell depletion is critical for consolidating treatment responses and achieving long-term disease control. Importantly, antibody clearance generally preceded proteinuria improvement by several months, suggesting that delayed remission reflects the time required for podocyte recovery, rather than true treatment resistance. However, these findings should be interpreted with caution. The observed numerical benefit of retreatment is likely attributable to the selective administration of a second course to higher-risk patients, rather than representing a universal causal benefit. Since the decision to retreat was non-randomized—driven largely by financial constraints rather than purely clinical judgment—the analysis carries potential selection bias. Therefore, while our data strongly supports a practical, individualized strategy that guides retreatment decisions based on combined antibody trends and proteinuria levels, rather than following a fixed schedule, these findings remain hypothesis-generating and require further validation in prospective randomized trials.

Regarding safety, the combination of low-dose corticosteroids and RTX was well tolerated by most patients: adverse events (AEs) occurred in 37.1% of cases, and serious adverse events (SAEs) occurred in 11.4% (4 out of 35 patients). The incidence of AEs in our study was lower than the 50%–80% rate reported in previous literature (Fervenza et al., 2008), while the incidence of SAEs fell within the previously reported range of 0%–17% (Fervenza et al., 2010; Cravedi et al., 2007). This lower AE rate we observed may be attributable to preemptive antihistamine administration and controlled infusion rates, which mitigated infusion-related reactions (Courville et al., 2021; Fouda and Bavbek, 2020). All four SAEs recorded in our study were infection-related: one case of cytomegalovirus (CMV) infection and three cases of pulmonary infection. Although we implemented standardized prophylactic measures, including trimethoprim-sulfamethoxazole prophylaxis, entecavir for hepatitis B core antibody (HBcAb)-positive patients, and serial CMV IgG monitoring, these infection events still highlight the inherent infection risk of B-cell depletion therapy combined with glucocorticoids. Importantly, retrospective medical record review identified no cases of severe glucocorticoid-specific toxicities, such as psychosis, peptic ulcer disease, or avascular necrosis, which supports the good tolerability of the low-dose steroid regimen. All SAEs were rigorously verified, and no malignant or fatal events were reported. These findings underscore the need for careful infection surveillance when rituximab is combined with glucocorticoids.

This study has several limitations. First, the generalizability of our findings is restricted by the single-center design, relatively small sample size (n = 35), and specific baseline characteristics of our cohort: specifically, we included a low proportion of patients with severe renal dysfunction (eGFR <60 mL/min), high-titer anti-PLA2R antibodies (>150 RU/mL), or prior cyclophosphamide exposure. Additionally, the absence of THSD7A and NELL-1 antibody testing means we cannot rule out rare secondary membranous nephropathy subtypes or dual seronegativity, which may have affected the accuracy of our treatment response assessment. Second, potential confounding factors include the fact that two-thirds of our patients had received recent prior immunosuppressive therapy, as well as time-dependent bias introduced by the prespecified protocol rule that a second rituximab course would be administered based on interim proteinuria response. Third, the granularity of our safety assessment was limited: although medical record reviews found no severe glucocorticoid-induced complications (such as psychosis, peptic ulcers, or avascular necrosis), steroid toxicities were managed according to routine clinical practice rather than systematically graded by CTCAE criteria, which likely led to mild adverse effects such as transient hyperglycemia being underestimated. Fourth, while we achieved the original sample-size target for the prespecified 12-month endpoint, only 22 patients had evaluable data for the revised 24-month endpoint. This smaller sample size, combined with higher-than-anticipated attrition, reduced the statistical power and precision of the 24-month analysis; therefore, these findings should be interpreted with caution. Fifth, because this is an uncontrolled cohort study, we cannot draw causal conclusions about the incremental benefit of adding glucocorticoids to rituximab treatment. Our empirically designed regimen means that the observed high remission rate may be attributable to repeated rituximab exposure and prior immunosuppression rather than the direct effect of glucocorticoids, so larger randomized controlled trials are required to confirm these findings. Finally, the median follow-up duration of 24 months is not long enough to capture late relapses, rare late-onset toxicities (such as avascular necrosis and malignancy), or progression to end-stage renal disease; longer-term follow-up is needed to verify the durability of both treatment efficacy and safety.

## Conclusion

In this cohort of refractory primary membranous nephropathy, rituximab combined with low-dose glucocorticoids achieved high short-term remission rates with no safety signals that would preclude further investigation. However, cautious interpretation is warranted due to the small sample size, infection-related serious adverse events, lack of systematic steroid toxicity grading, and limited follow-up duration. These hypothesis-generating findings require validation in future large-scale, multicenter controlled trials incorporating B-cell-guided therapy, quality-of-life assessments, and systematic monitoring of long-term complications such as weight gain and osteoporosis.

## Data Availability

The data that support the findings of this study are available from the corresponding author upon reasonable request.

## Supplementary material

SUPPLEMENTARY FIGURE 1 Longitudinal changes in clinical, biochemical, and immunological parameters following rituximab treatment. Mixed-effects model marginal means with 95% confidence intervals (shaded areas) for **(A)** serum albumin; **(B)** log-transformed proteinuria; **(C)** total cholesterol; **(D)** triglycerides; **(E)** estimated glomerular filtration rate (eGFR); **(F)** serum immunoglobulin G (IgG); **(G)** log-transformed anti-PLA2R antibodies; **(H)** log-transformed CD19+ B cells. Gray lines represent individual patient trajectories, and the orange line represents model-estimated marginal mean. Note: y-axes for panels B, G, and H are log-transformed to improve visualization of distribution.

SUPPLEMENTARY FIGURE 2 Anti-PLA2R antibody levels at baseline and Month 6 in 23 patients receiving a second rituximab course. The data show a marked reduction from baseline (median 60 [IQR 20–100] RU/mL) to Month 6 (median 7.22 [IQR 5.17–11.45] RU/mL) in the cohort receiving a second infusion.
