# Supplementary material for: Rituximab with low-dose glucocorticoids for treating refractory membranous nephropathy: a prospective single-center cohort study
Source: Front Pharmacol. 2026 Sep 25;17:1894444. doi: 10.3389/fphar.2026.1894444 (PMC13649750; doi:10.3389/fphar.2026.1894444)
Supplement: Supplementary file 1 [file Supplementaryfile1.docx]

**Supplementary Table 1. Characteristics of prior immunosuppressive therapies before rituximab initiation**

| Medication Class | Exposure  [n (%)] | Duration  Median (IQR), mos | Daily Dose Median (IQR) | Best Response to Prior Tx^a^ [n (%)] | Washout Period^b^ Median (IQR), wks |
| --- | --- | --- | --- | --- | --- |
| Calcineurin Inhibitors | 28 | 9 (6,12) | / | CR: 2 (7.1) PR: 12 (42.9) NR: 14 (50.0) | / |
| Tacrolimus | 22 | 10 (6, 14) | 2.0 (2.0, 2.5) mg/d | CR: 1 (4.5) PR: 9 (41.0) NR: 12 (54.5) | 4 (2.5, 18) |
| Cyclosporine | 18 | 8.5 (5.8, 12) | 150(150,181.3) mg/d | CR: 1 (5.6) PR: 8 (44.4) NR: 9 (50) | 11 (4, 31) |
| Cyclophosphamide | 9 | 6 (5, 9.5) | 0.8 (0.7, 0.8) g/mo | CR: 1 (11.1) PR: 4 (44.4) NR: 4 (44.4) | 48 (10, 74) |
| MMF | 4 | 8 (4.5, 16) | 2.0 (1.75, 2.0) g/d | NR: 6 (100) | 9 (4.5, 39) |
| Tripterygium wilfordii | 5 | 9 (1.5, 14) | 60 (50, 60) mg/d | PR: 1 (20) NR: 4 (80) | 56 (28, 246) |
| Leflunomide | 2 | 5 (4, 6) | 20 (20, 20) mg/d | NR: 2 (100) | 14 (4, 24) |

a: Response definitions: CR, complete remission (proteinuria < 0.3 g/d); PR, partial remission (proteinuria reduction ≥ 50% and < 3.5 g/d); NR, no response.

b: Washout period defined as the interval between discontinuation of prior agent and the first RTX infusion. NA: Not applicable for corticosteroids as they were typically tapered but continued.

Note on overlap: Percentages sum exceeds 100% due to sequential or combination therapies (e.g., Tacrolimus + MMF).

**Supplementary Table 2. Characteristics of the study cohort and rituximab dosing distribution**

|  | Single Course Only (n=12) | Two Courses (n=23） | Total Cohort (n=35) | ***P* value** |
| --- | --- | --- | --- | --- |
| Cumulative RTX dose [g, median (IQR)] | 2 (2, 2) | 4 (4, 4) | 4 (2, 4) | <0.0001 |
| Indication for re-treatment [n (%)] |  |  |  |  |
| Proteinuria and PLA2R persistence | 2 (16.7） | 4 (17.4) | 6 (17.1) | 1 |
| Proteinuria only | 8 (66.7) | 19 (82.6) | 27 (77.1) | 0.402 |
| PLA2R only | 0 | 0 | 0 | 1 |
| B-cell reconstitution at M6  [n (%)] | 3 (25.0) | 10 (43.5) | 13 (37.1) | 0.463 |
| 24-h proteinuria at M6 [g, median (IQR)] | 3.05(1.03,7.84） | 1.84 (1.03，4.12) | 1.92 (1.11，5.31) | 0.504 |
| PLA2R titer at M6 [RU/mL, median (IQR)] | 6.94 (5.00, 17.87) | 7.22 (5.17，11.45) | 7.08 (5.04，11.16) | 0.792 |
| Remission rate at 12 months [n (%)] | 6 (50) | 20 (87) | 26 (72.2) | 0.037 |

Data are presented as median (interquartile range, IQR) or number (percentage). M6, Month 6; RTX, rituximab; PLA2R, phospholipase A2 receptor.

Abbreviations: IQR, interquartile range; M6, Month 6; RTX, rituximab; PLA2R, phospholipase A2 receptor.

**Supplementary Table 3. Clinical outcomes of patients who declined versus those who received a second course of rituximab.**

| **Outcome** | **Declined second course (n=6)** | **Received second course (n=23)** |
| --- | --- | --- |
| **12-month overall remission[ n (%)]** | 5 (83.3%) | 18 (78.3%) |
| CR | 3 | 6 |
| PR | 2 | 12 |
| **Median time to remission (months)** | 4 | 6 |
| **24-month remission among completers[ n (%)]** | 2/4 (50.0%) | 17/18 (94.4%) |
| CR | 1 | 9 |
| PR | 1 | 8 |

Data are presented as n (%) unless otherwise specified. The retreated cohort included 23 patients who received a second course, of whom 18 completed the 24-month follow-up. The declined cohort included 6 patients, of whom 4 completed the 24-month follow-up.
